# Obesity and bariatric surgery in adults living with severe mental illness: perceptions and clinical challenges

**DOI:** 10.1192/bjb.2022.31

**Published:** 2023-06

**Authors:** Carolina Pressanto, Samantha Scholtz, Nasteha Ali

**Affiliations:** 1 Kent and Medway NHS and Social Care Partnership Trust, UK; 2 St Mary's Hospital, Imperial College Healthcare NHS Trust, UK; 3 West London NHS Trust, UK

**Keywords:** Antipsychotics, bipolar affective disorders, comorbidity, schizophrenia, psychotic disorders

## Abstract

Overweight and obesity are twice as likely to develop in people living with severe mental illness (SMI), compared with those without. Many factors contribute to this, such as reduced physical activity and the use of certain medications that induce weight gain. Obesity contributes to the premature mortality seen in people living with SMI, as it is one of the fundamental risk factors for cardiovascular disease and diabetes. Bariatric surgery is an effective treatment option, although patients living with SMI might face stigma when being considered for surgical intervention. This article proposes a discussion around obesity and bariatric surgery in patients living with SMI. It will also reflect on the challenges faced by healthcare professionals and patients living with SMI and obesity, when considering appropriate treatments for weight loss. The paper utilises a fictional case, informed by contributions from a lived experience author, to explore bariatric surgery in people living with SMI.

## Clinical scenario

Lilian is a 43-year-old female who has a follow-up appointment with the community mental health team where you are currently working as a core trainee. You know from her records that she was diagnosed with bipolar affective disorder at age 30 years, and had a psychotic episode at age 35 years. She has been on clozapine and lithium since then, which she takes regularly. During the consultation, she mentions to you she has gradually gained weight since adolescence and it has now reached a point that she is worried about the stability of her mental illness being affected by her eating patterns and consequent weight gain, as if it was ‘a vicious cycle’. For instance, the more weight she gains, the worse she feels about herself. Feeling bad about herself makes her depression worse, and when she is depressed, she does not want to get out of bed and does not see the point in anything, to the point that both healthy eating and exercise regimes seem out of reach. She also says to you that she noticed a considerable increase in appetite after starting the antipsychotics and consequent weight gain. She describes that she now eats to fill a void sensation inside her that she does not completely understand.

Questions*:*

- What is the relationship between obesity and SMI?
- How should clinicians approach conversations about weight gain and the clinical assessment of factors contributing to obesity?
- What comorbidities need to be considered?

## Relevance of obesity in SMI

The prevalence of obesity is increasing worldwide, and has nearly tripled since 1975.^1^ In England, 67% of men and 60% of women were classified as overweight or living with obesity in 2018.^2^ Although the causes are multifactorial, there is a clear positive association between lower socioeconomic level and obesity.^3^ The most commonly used method to assess health risks associated with high body weight is body mass index (BMI). In adults, obesity is defined as a BMI ≥30 kg/m^2^.^1,4^ A BMI of >30 kg/m^2^ is a major risk factor for cardiovascular disease, diabetes and cancer, among other non-communicable diseases.^1^

In those living with severe mental illness (SMI) such as schizophrenia and bipolar affective disorder, there are high prevalence rates of obesity and metabolic disorder (40–50% and 50%, respectively).^5^ Additionally, people living with SMI have significantly lower life expectancy than the general population. A study from a secondary mental healthcare case register in London with 31 719 participants showed a reduction of approximately 12 years in life expectancy at birth in people living with any SMI.^6^ Other studies have shown the same pattern of premature death and decades of potential life lost (averages varying from 13 to 30 years) among people living with SMI.^6,7^ Underlying causes for raised mortality in SMI may be multiple. Leading causes of death are cardiovascular disease, unintentional injuries and suicide. Obesity, hypertension, diabetes mellitus and chronic obstructive pulmonary disease are among the most prevalent medical comorbidities in this population. Factors likely to contribute to the development of these conditions are increased prevalence of smoking, increased substance use, inadequate social support, reduced physical activity and medication-induced weight gain.^7,8^

Some antipsychotics might play a significant role in the development of metabolic syndrome, as they might cause glucose dysregulation, weight gain and lipid disturbance. The most efficacious antipsychotics, such as clozapine and olanzapine, seem to be associated with more weight gain and metabolic syndrome risk.^9,10^ Weight gain associated with antipsychotics can discourage patients from consistently taking their medication and, consequently, increase the chances of relapse. It is therefore important to have open and honest discussions with patients about these side-effects during the consultation. A non-judgemental approach is paramount to establish good rapport with patients. People who struggle with losing weight are often left with feelings of guilt and shame, as they feel blamed by healthcare practitioners. This can be a significant contributor to internalised stigma, which is known to reduce success with weight loss interventions and can negatively affect patients’ coping abilities and motivation to lose weight. Healthcare practitioners should be aware of the biological drivers to weight regain that make long-term weight loss difficult to achieve without bariatric surgery.^11–13^ Implementation of healthy weight management strategies as early as possible for individuals who are taking antipsychotic medication is also essential.^14^ People living with SMI benefit from lifestyle changes targeting to reduce body weight, such as weight management counselling and group exercise, despite the use of psychotropic medication and ongoing psychiatric symptoms – factors which could potentially affect adherence to such methods.^15^ Liraglutide, a glucagon-like peptide-1 receptor agonist, is associated with reduced body weight, improved glucose tolerance and reduction in systolic blood pressure and low-density lipoprotein levels, when compared with placebo, in patients living with SMI who are using antipsychotics.^16^

In addition, the accumulation of adipose tissue can influence hormonal and biochemical responses. Increased abdominal adiposity seems to be related to a greater response of the hypothalamic-pituitary-adrenal (HPA) axis and accumulation of cortisol in adipocytes.^17^ Social circumstances are also important contributing factors. Adverse childhood experiences, such as physical and sexual abuse, exposure to domestic violence and household mental illness, are strong predictors for development of health issues later in life. This includes heavy alcohol use, heart disease, cancer, obesity and mental ill health (including depression, anxiety and suicide attempt).^18^ These early-life stressors might also trigger the HPA axis, which can lead to metabolic changes.

## Exploring root causes: take-home message for your clinical consultation

Understanding the mechanisms that drive people living with SMI to gain weight is a process that requires time and active listening, as it is often a non-linear path with periods of satisfactory weight control intercalated with periods of overeating. It is important to identify unhealthy eating patterns as a form of coping mechanism for stressful situations. Once this is recognised and acknowledged by the patient, it becomes an opportunity to find possible root causes and triggers, dissect these issues and explore alternative healthier coping mechanisms. During the consultation, assess the person's view of possible reasons for weight gain, their readiness to adopt changes and their confidence in making those changes. Ask about comorbidities (such as type 2 diabetes, cardiovascular disease, hypertension) and explore any psychological issues and eating behaviours,^4^ in view of screening for factors that might affect the success of treatment, such as alcohol misuse, intellectual disability and eating disorders (including binge eating disorder).

### Clinical scenario

During the appointment, you address Lilian's concerns over her eating patterns and weight gain, and she confides to you that a male family member had sexually abused her when she was 13 years old. The abuse lasted for 4 years and she remembers putting on a significant amount of weight during that time, which she believes was a coping mechanism for what she was going through as well as an attempt to attract less attention from her abuser. She recalls using food as a way to escape unpleasant thoughts and numb her feelings. Lilian said her relationship with food changed completely in that time, when she began to see food as a comfort in difficult times. This pattern continued through adulthood, when she used to overeat when she faced herself in challenging situations, such as her younger sister passing away after a car accident. Since she was 20 years old, she has been in a series of relationships with men that physically and psychologically abused her, and she identified these unhappy relationships as triggers for overeating. Ever since her previous psychiatrist has started her on antipsychotics, she noticed a significant weight gain that, after having researched about side-effects, she attributed to the use of those medications. Lilian says that she would feel hungry again 30 min after finishing a meal, in which she would feel a strong compulsion to eat again. Over the past couple of years, she has engaged in diet changes and exercise programmes, although she has not been able to lose weight. These interventions have been affected by the COVID-19 pandemic, in which Lilian stopped attending the gym because of the implemented restrictions. She also found social isolation brought about by the lockdown rules particularly difficult to cope with, as she was not able to attend face-to-face groups and it was challenging to establish a direct line of communication with the healthcare system. She recognised that she again reached for food as comfort and company in these uncertain times. Lilian feels that her self-esteem has been greatly affected by all this, which makes it harder for her to continue with weight loss programmes. She now feels hopeless around her continuous weight gain, as if she was not in control of it. Lilian understands and agrees she still uses food as a comfort measure. However, she does not know what else to do in those moments when she feels as if there is something missing inside of her and she knows that food will fill that gap by bringing her comfort. The more she thinks about this, the more distressed she gets and, consequently, the more she eats.

Questions:

- What are the available treatment options for people with obesity and what adjustments or considerations might be required for people living with SMI?
- When should referral for bariatric surgery be considered?
- How is the assessment for bariatric surgery done?

## Treatment options for obesity

**Fig. 1:**
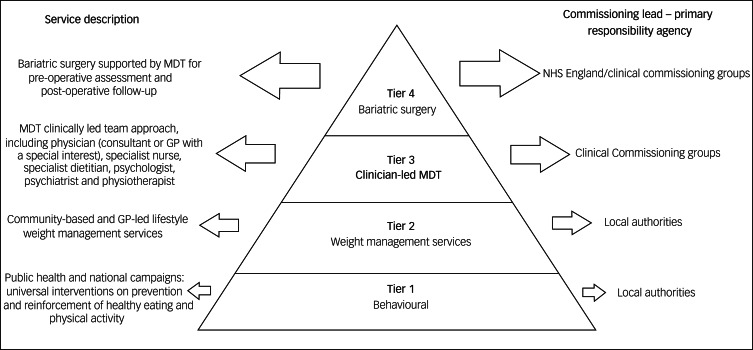
Definition of the tiers and comissioning lead for weight management. Based on the Commissioning guidance for weight assessment and management in adults and children with severe complex obesity, 2018. (GP, general practitioner; MDT, multidisciplinary team)

Considering all treatment options for obesity for people living with SMI is important, particularly when we contemplate the potential positive influence on quality of life that these interventions can offer. The National Institute for Health and Care Excellence (NICE) has outlined interventions according to a four-tier system (see Fig. 1), which consists of universal interventions, lifestyle weight management services, multidisciplinary clinically led team approach and, finally, bariatric surgery services. The choice of intervention should be agreed with the patient.^19^ Lifestyle and behavioural interventions, as well as dietary and physical activity advice, are the base of treatment. One study conducted with patients living with SMI who had raised BMI and were participating in psychiatric rehabilitation programmes evidenced significant reduced weight in patients undergoing weight management counselling and group exercise over a period of 18 months. Despite psychotropic medication use and ongoing psychiatric symptoms, patients in the intervention group had a mean change in weight from baseline of −3.4 kg, compared with −0.2 kg in the control group at 18 months.^15^ Pharmacological intervention is considered after dietary, exercise and behavioural changes were not sufficient to reach the targeted weight loss.^4^ The current licensed drugs are orlistat and liraglutide. The first is recommended for people with BMI >30 kg/m^2^ or BMI >28 kg/m^2^ with risk factors. The second can be prescribed by a physician as part of a tier 3 specialist weight management service, and it is intended for people with BMI of at least 35 kg/m^2^ or at least 32.5 kg/m^2^ for those of higher-risk ethnic groups, presence of non-diabetic hyperglycaemia and high risk of cardiovascular disease.^19,20^ For people taking antipsychotics who experience weight gain and where non-pharmacological interventions/changing antipsychotic agents are not sufficient measures to reduce weight, metformin is the first choice of drug. It has positive effects in body weight, insulin resistance and lipid profile in people who are prescribed antipsychotic medication.^21^

Bariatric surgery is considered when BMI >35 kg/m^2^ and comorbidities are present (such as type 2 diabetes and hypertension) or BMI >40 kg/m^2^ without comorbidities. If the patient has recent-onset type 2 diabetes and a BMI of 30–34.9 kg/m^2^, consider an assessment for bariatric surgery. Clinicians should also consider assessment for bariatric surgery for people with Asian family origin who have recent-onset type 2 diabetes at a lower BMI than other populations. Other inclusion criteria are commitment to the need of long-term follow-up and intensive management in a tier 3 service.^22^ Bariatric procedures result in greater weight loss than other forms of medical treatment for obesity, and can reduce incidence of diabetes,^23^ potentially contributing to reducing cardiovascular risk factors.

Surgery for obesity should be undertaken by a multidisciplinary team that can provide a comprehensive pre-operative assessment (risk–benefit analysis, specialist assessment for eating disorders), psychological support and management of comorbidities.^22^ Clinicians should consider having a discussion with the mental health team around the patient's understanding of bariatric surgery, how they would cope in a tier 3/4 environment and what further support they would require after surgery. When referring to tier 2, 3 and 4 services, clinicians should be attentive to the patient's mental health needs that might take priority at that point or prevent them from engaging in treatments. However people living with SMI can do as well with community-led interventions as those without.^24^ Care should be taken to guard against the risk of diagnostic overshadowing, where obesity is missed or not attended to because of the presence of SMI. General practitioners should progress through the tiers as soon as possible, being mindful that patient's poor engagement with services should not be an impediment to refer to tier 3 services. Usually, there is no exclusion criteria for referral for tier 2 services, although clinicians should avoid referring during a mental health crisis.

Although psychological support is included in NICE guidelines recommendations, guidelines for the provision of psychological support pre- and post-bariatric surgery has been difficult to implement because of a lack of resources. In 2019, the British Obesity Metabolic Surgery Society endorsed guideline suggests a stepped-care approach involving online resources, group workshops and one-to-one contact with a clinical psychologist. Which one of the three steps will be used is based on the issue presented by the patient and the potential impact if the issue is not addressed. It is recommended that all those seeking bariatric surgery have a psychological assessment triage, to identify the support they require to optimise weight loss and psychological outcomes.^25^

### Clinical scenario

Lilian was eventually referred to the bariatric centre and assessed by the multidisciplinary team, who deemed her fit to proceed with bariatric surgery. Following surgery, Lilian attended the accident and emergency department a few times, reporting dizziness after eating. Investigations were negative and Lilian was reassured by the accident and emergency team that she was having late dumping syndrome symptoms (reactive hypoglycaemia), and that this can occur in a number of patients after bariatric surgery. She was given general advice, such as eating regular meals and avoiding sugary drinks and foods, and referred to a specialist dietitian for further advice and support. She was under the care of a multidisciplinary team for 2 years, including a dietitian and psychologist, to help her navigate through the changes that bariatric surgery had brought to her life, but they have now discharged her from their care and advised her to visit her general practitioner once a year for check-ups.

Three years on and Lilian has an appointment in the community mental health centre that you are now returning as a higher trainee. She tells you she is managing quite well overall, feeling happy about having lost weight and consistently maintaining it, and it has positively affected her mental health.

Questions:

- What is involved in follow-up for bariatric surgery?
- What is the prognosis of people with SMI who have bariatric surgery?

## Follow-up after surgery

People who had bariatric surgery must be offered a follow-up package for a minimum of 2 years within the bariatric service, which should include support on diet, nutrition, physical activity and psychological support.^22,26^ It is recommended that all patients have a psychological screening assessment at 6 and 9 months post-surgery, applying the previously mentioned stepped-care approach to address any issues identified, although this is practically difficult to implement because of a lack of resources.^25^ In people living with SMI, where the bariatric service does not have its own psychological support in place, planning post-operative review by the mental health team may support picking up post-operative issues if they arise. After discharge from bariatric surgery service, all patients should be offered annual monitoring in a shared-care model of chronic disease management.^22,26^

A large retrospective cohort study involving 8000 patients was published in 2017, with the aim to evaluate if patients living with mental illness, particularly SMI, would have less weight loss and higher post-operative acute care use after bariatric surgery compared with patients with no mental illness. In this study, 57% of the patients selected had a mental health diagnosis in their records (before pre-operative evaluation) and the median follow-up was 2.9 years. Results from this large study showed that patients living with mental illness, even those living with SMI, had very similar weight loss compared with those with no mental illness pre-operatively, suggesting that patients with a history of mental illness can benefit equally from surgical treatment of obesity. However, pre-operative diagnosis of mental illness was predictive of greater acute care use, although the authors did not examine cause-specific healthcare use after surgery.^27^

## Reflections and considerations

### From the doctor's perspective

Mental health professionals should aim to provide patients with relevant information regarding common side-effects of psychotropic medication in an open dialogue situation, as well as offer support measures in the form of targeted weight loss interventions for this high-risk population. Patients living with SMI and obesity are at risk of double stigmatisation. Bariatric surgery is an effective treatment for obesity, and we should consider this option for people living with SMI. Psychological support and multidisciplinary approach are key to understand and support patients in the complexity of their setting, especially when it comes to understanding eating patterns, teaching new tools to be used as healthy coping mechanisms and helping patients understand their own condition. We should encourage patients to participate in the decision-making process and become equally responsible for their health and well-being, making use of a non-judgemental approach. In terms of follow-up after surgical procedure, the multidisciplinary team must be attentive to alternative harmful coping mechanisms that might develop even after the 2-year follow-up with the bariatric service, such as alcohol misuse.

### From the patient's perspective

There needs to be a frank and open dialogue between patients and doctors in charge of their treatment, to prepare patients for possible outcomes of antipsychotic use, using tools that are practical to help combat the medication/weight gain issue. Lilian, as with many patients living with SMI and obesity, may struggle with more than one form of long-term condition and with managing these. Patients may be reluctant to be completely open about negative symptoms, as they sometimes cannot link their motivation to eat with why this may be so. There is an additional fear of judgement when talking about their relationship with food. Treating Lilian holistically and teaching her good coping skills may help the feeling of being overwhelmed by something she has no way of fixing, and may support her with other aspects of her care, including self-management of her SMI. Often, patients that are waiting to have bariatric surgery might feel lost and in limbo, with no clear direction or personal responsibility, with a feeling of waiting until the day they have the surgery to start living again, without doing the necessary internal work to ensure they adapt a sustainable long-term change.

**Practical management**

- Be mindful of side-effects when prescribing antipsychotics, monitor weight gain – consider weighing the patient on follow-up appointments and offer support for weight loss: ‘Before you leave, could I check your weight today?’ ‘One of the best ways to lose weight is with support and [insert name of weight management service] is available for free. I can refer you now if you are willing to give it a go?’ (see recommended additional reading for further approach suggestions).
- Adopt a holistic approach when managing patients living with obesity and severe mental illness; understand the pathway that led them to gain weight and their relationship with food, screen for eating disorders and refer for treatment if identified. Support the person to develop alternative tools to be used in psychologically challenging situations.
- Explore a weight history – look at causal factors for obesity and associated comorbidities (such as type 2 diabetes, hypertension) and presence of eating disorders.
- Understand the physiological drivers to weight regain so that you approach all people living with obesity with respect and compassion.
- Treat obesity in persons living with severe mental illness according to current guidelines; consider tier 2, 3 and 4 level treatments and refer to bariatric surgery if non-surgical options have been tried.
- If bariatric surgery is recommended, explain to the patient what this involves (commitment to follow-up, engagement with bariatric services and side-effects after surgery, for instance) and explore with them their thoughts about it; actively involve patients in their treatment and support them in a shared decision-making process.

## Conclusion

People living with SMI and obesity face double stigmatisation. Both conditions are predictors of premature death. Clinicians should be aware of the effects of antipsychotics on weight gain, discuss this with patients and provide them tools in the form of targeted weight loss interventions. Bariatric surgery is an effective treatment option for obesity, results in significant weight loss, potentially contributing to improvement of cardiovascular risk factors, and it is equally effective in patients living with SMI. It is part of our role as psychiatrists to support patients living with SMI to have appropriate treatment for obesity. We should advocate for our patients to have bariatric surgery where appropriate, maintain an open and non-judgemental dialogue with patients and support them through this challenging process.

## Recommended additional reading

1. Teale KFH, Devine A, Stewart H, Harper NJH. The management of metformin overdose. *Anaesthesia* 2002; 53(7): 698–701.
2. Every-Palmer S, Romans SE, Stubbs R, Tomlinson A, Gandhi S, Huthwaite M. Experiences of weight-loss surgery in people with serious mental illness: a qualitative study. *Front Psychiatry* 2020; 11: 419.
3. Thompson L, Aveyard P, Jebb S, Blackshaw J, Coulton V, Tedstone A. *Let's Talk About Weight: A Step-By-Step Guide to Brief Interventions with Adults for Health and Care Professionals.* Public Health England, 2017 (https://assets.publishing.service.gov.uk/government/uploads/system/uploads/attachment_data/file/737903/weight_management_toolkit_Let_s_talk_about_weight.pdf).
4. Scarpellini E, Arts J, Karamanolis G, Laurenius A, Siquini W, Suzuki H, et al. International consensus on the diagnosis and management of dumping syndrome. *Nat Rev Endocrinol* 2020; 16(8): 448–66.

## About the authors

**Carolina Pressanto** is a Core Specialty Registrar in General Adult Community Mental Health Team with Kent and Medway NHS and Social Care Partnership Trust, UK. **Samantha Scholtz** is a Consultant Psychiatrist in bariatric surgery at the Imperial Weight Centre, St Mary's Hospital, Imperial College Healthcare NHS Trust, UK; and Research and Development Director at the West London NHS Trust, UK. **Nasteha Ali** is a lived experience author and a Patient and Public Representative at the R&D department, West London NHS Trust, UK.
